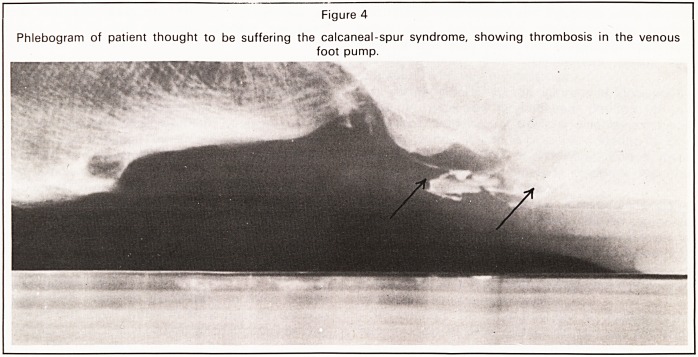# The Venous Pump of the Human Foot—Preliminary Report

**Published:** 1983-07

**Authors:** A. M. N. Gardner

**Affiliations:** Torbay Hospital


### The Venous Pump of the Human Foot—Preliminary report

**A. M. N. Gardner, D.M., M.Ch., F.R.C.S. and R. H. Fox, F.R.C.R.**
Torbay Hospital

We have discovered a venous pump mechanism in the sole of the human foot that is able to return blood from the leg up into the abdomen with no assistance from muscular action.

The veins of the sole of the foot have been regarded as insignificant from the pathological point of view[1] and most anatomical descriptions refer merely to medial and lateral plantar arches although French anatomists have referred to the 'semelle veineux plantaire' or 'le reseau veineux plantaire profund'—the dense plexus or insole of veins or the deep plantar venous network[2] but conventional anatomical diagrams bear little relation to the large calibre veins as demonstrated by orthostatic phlebography.[3]

If little interest has been shown in the venous anatomy of the foot, less has been shown in the physiology of venous return. Although surgeons have realised that squeezing the foot in the supine position increases flow in the normal femoral vein as judged by Doppler as does compression of calf or thigh; (this test is used to show patency or otherwise of the femoral veins). It has been vaguely assumed that some pump system exists activated probably by ankle or toe movements and rise in venous pressure on weight bearing has been recorded.[4] It is surprising that there is so little basic knowledge of the vascular system of the foot particularly in view of the clinical importance of flat foot, and the plantar fasciitis-calcaneal spur syndrome.

If little attention has been paid to anatomy and physiology of the veins of the foot, even less has been paid to the venous pathology. Morbid anatomists dissect the foot only when obviously diseased, in any case the present climate of opinion is opposed to such routine dissection since it could lead to accusations of unnecessary mutilation of the body. For this reason primary venous thrombosis has not been previously described.

We studied the venous return of the normal foot of one of us (A. M. N. G.): contrast medium, Hexabrix (May & Baker Ltd), was injected into a vein on the dorsum of the foot while standing and weight bearing only on the other foot. Flow of contrast was recorded by video-phlebography and intermittent conventional radiography with synchronous observer commentary. The contrast agent was seen to flow via a channel between the second and third metatarsal bases and to pool in two deep medial plantar veins and in their more superficial tributaries, the most posterior of these being large and plexiform (Figures 1 and 2). Neither toe nor ankle movements

**Figure 1**

Phlebogram of normal non-weight bearing foot. Antero-posterior View.

**Figure 2**

Phlebogram of same non-weight bearing foot. Lateral view.

influenced the plantar pool of contrast in this non-weight bearing foot, but as soon as weight was borne, the deep plantar veins emptied up into the calf. Weight bearing on a narrow transverse pad under the instep emptied mainly the deep plantar veins in that immediate area whereas weight bearing simultaneously on heel and metatarsal heads emptied the whole system (Figure 3). These findings were confirmed in two further volunteers and in the normal feet of two patients with contra-lateral venous problems.

Flow up the deep veins of the leg on weight bearing was confirmed by ultrasonic velocimetry and it was found that weight bearing on the foot with all leg muscles immobile and tensed to the maximum by voluntary contraction (ten observations), still caused upward flow in the femoral vein at the groin. Likewise weight bearing on a flaccid hemiplegic leg with the knee locked, also caused flow in the femoral vein. (Two observations.)

In constrast to the radiographic findings in the foot, venous return from the calf is certainly influenced by active orthostatic ankle movements, but in the opposite way to that generally accepted[5] in that the deep intermuscular veins in the lower calf are clearly seen on video-phlebography to empty upwards or dorsiflexion of the ankle whereas on plantar flexion these same veins fill from the superficial long saphenous vein via the veins that perforate the fascial envelope of the calf. Plantar flexion however does also propel blood upwards as shown by Doppler test on the femoral vein in the groin. This flow probably originates from the intramuscular venous sinuses since these are compressed during contraction of the posterior calf muscles.[6]

The venous 'foot pump' like all other organs in the body, is likely to suffer various disorders of structure and function and indeed we have already demonstrated intra-vascular thrombi in the 'foot pump' of a patient suspected clinically to be suffering from plantar fasciitis (figure 4); it seems therefore that plantar venous thrombosis is at least one cause of this puzzling clinical syndrome.

There are a number of reasons why the venous footpump has not previously been detected: Firstly the plantar veins are embedded in dense fibro-fatty tissue and in conventional anatomical dissections do not appear in any way remarkable. They have not been properly described in anatomical texts although Leonardo da Vinci correctly illustrated the deep medial plantar vein which in his drawing was naturally in a collapsed state.[7]

Secondly, conventional phlebography with serial exposures of film gives little information on the

**Figure 3**

Phlebogram shows that weight-bearing has emptied the large plantar veins shown in Figure 2.

Figure 4

Phlebogram of patient thought to be suffering the calcaneal-spur syndrome, showing thrombosis in the venous foot pump.

dynamics of venous return. Cineradiography has not apparently been used in this field, but now the recent development of video-phlebography makes inexpensive dynamic studies possible with synchronised observer commentary.

Thirdly, in clinical practice phlebography has seldom if ever been performed in the upright position.

Fourthly, until the introduction of modern non-irritant radiographic contrast media their injection could cause considerable pain and this together with the risk of contrast induced phlebitis[8] made it unethical to examine normal limbs in this way.

Lastly, the advent of Doppler ultrasonic velocimetry has enabled non-invasive venous flow studies to be performed in conjunction with dynamic phlebography to assist in its interpretation.

Recognition of the importance of the venous footpump can be expected to influence treatment of leg fractures, injuries that are known always to cause venous thrombosis and sometimes permanent peripheral venous hypertension; it is also likely to result in more scientific assessment and treatment of varicose veins, the post phlebitic syndrome and the plantar fasciitis-calcaneal spur syndrome and to new methods for the prevention of thrombo-embolism.

### REFERENCES

1. MAY, R. (1979) Major problems in clinical surgery veins in the foot. **23**, 1–36.
2. WINCKLER, G. (1923) Les Veines Du Pied, *Arch. anat.* (Strasbourg) **37**, 175–184.
3. CHERMET, J. (1982) Atlas of phlebography of the lower limbs including the iliac veins. Series in Radiology Vol. 6. p. 37 Martinus Nijhoff.
4. FEGAN, G. (1967) Varicose veins. Compression sclerotherapy. Heinemann monograph, p. 31. London, Whitefriars Press Ltd.
5. DODD, H., COCKETT, F. B. (1976) The pathology and surgery of the veins of the lower limb. Edinburgh, London and New York, Churchill Livingstone, p. 53.
6. ALMEN, T., NYLANDER, G. (1962) Serial phlebography of the normal lower limb during muscular contraction and relaxation. *Acter. radiol. (Stockh)* **57**, 264.
7. LEONARDO DA VINCI (Cir. 1515) Anatomical drawings at Windsor Castle. Kenneth Clark Vol. III 19010. Recto. Phaidon 1969.
8. ALBREGHTSSON, U., OLSSON, C. G. (1976) Thrombotic side effects of lower limb phlebography. *Lancet* **1**, 723.

## Figures and Tables

**Figure 1 f1:**
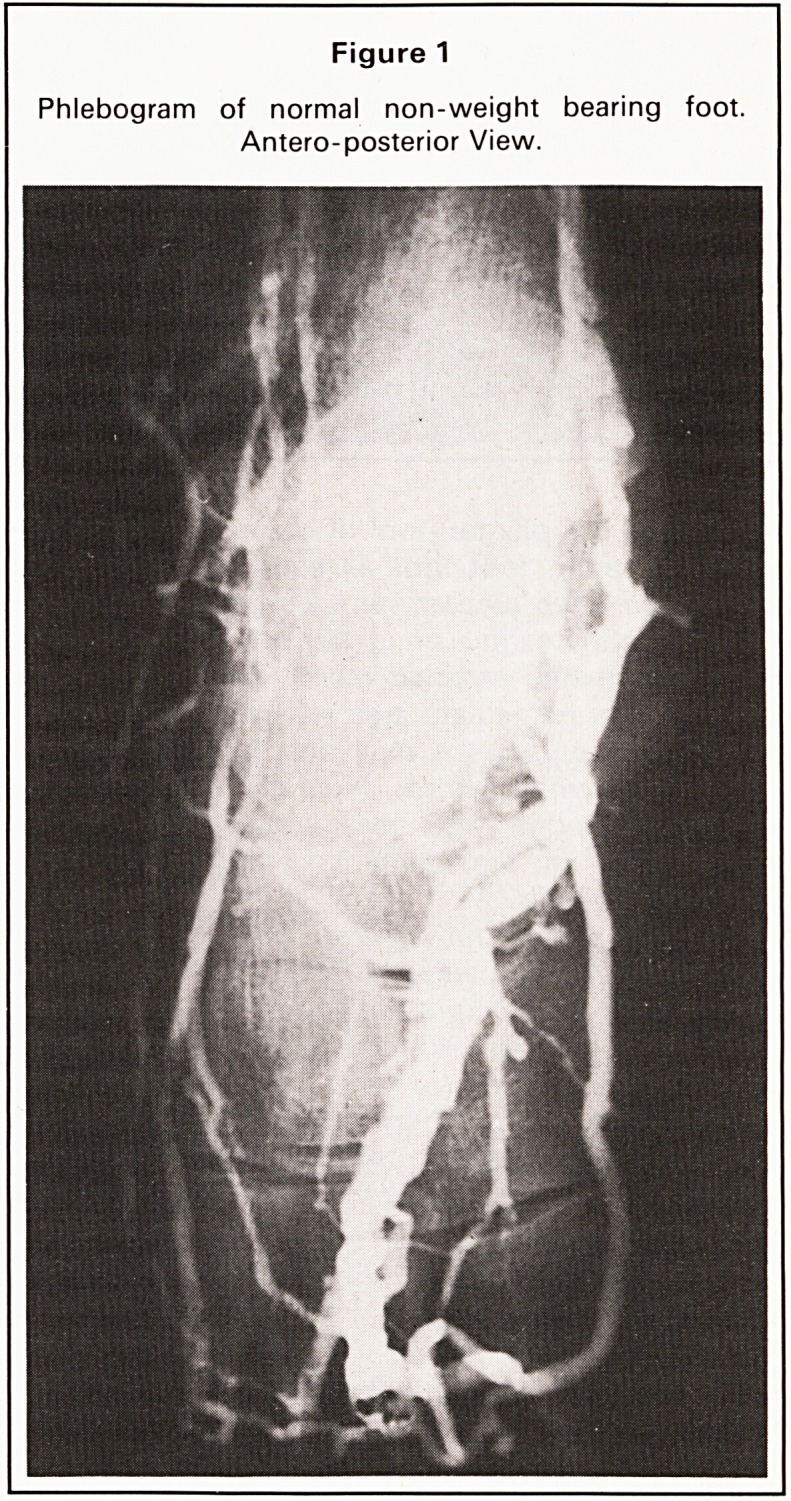


**Figure 2 f2:**
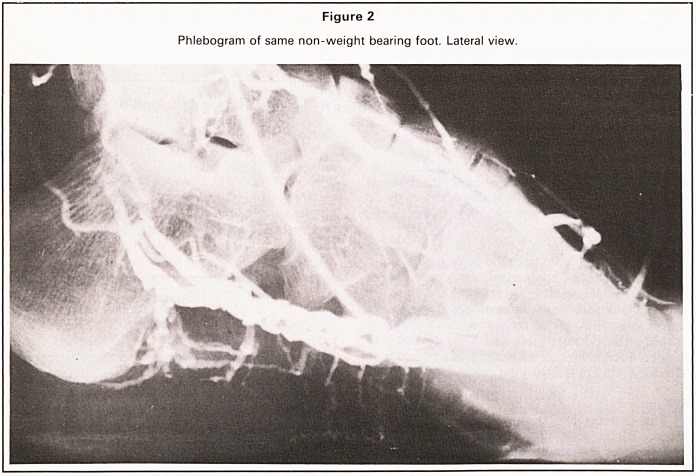


**Figure 3 f3:**
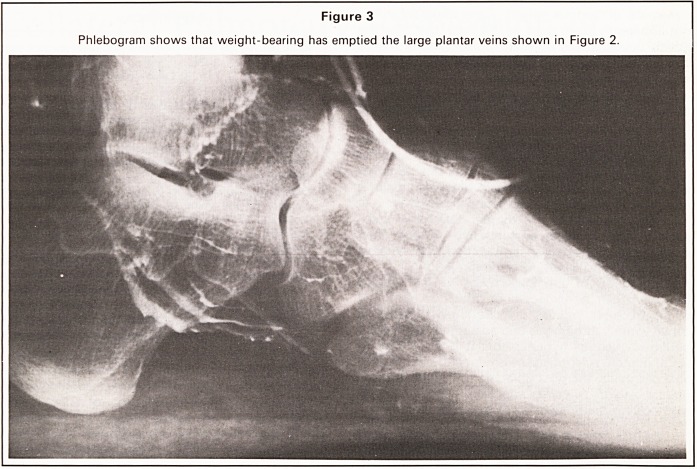


**Figure 4 f4:**